# Depression and Cognitive Impairment—Extrahepatic Manifestations of NAFLD and NASH

**DOI:** 10.3390/biomedicines8070229

**Published:** 2020-07-21

**Authors:** Martina Colognesi, Daniela Gabbia, Sara De Martin

**Affiliations:** Department of Pharmaceutical and Pharmacological Sciences, University of Padova, L.go Meneghetti 2, 35131 Padova, Italy; martina.colognesi@studenti.unipd.it (M.C.); daniela.gabbia@unipd.it (D.G.)

**Keywords:** non-alcoholic fatty liver disease, NAFLD, steatohepatitis, NASH, cognitive impairment, memory dysfunction, Alzheimer’s disease, neurodegeneration

## Abstract

Non-alcoholic fatty liver disease (NAFLD) and its complication non-alcoholic steatohepatitis (NASH) are important causes of liver disease worldwide. Recently, a significant association between these hepatic diseases and different central nervous system (CNS) disorders has been observed in an increasing number of patients. NAFLD-related CNS dysfunctions include cognitive impairment, hippocampal-dependent memory impairment, and mood imbalances (in particular, depression and anxiety). This review aims at summarizing the main correlations observed between NAFLD development and these CNS dysfunctions, focusing on the studies investigating the mechanism(s) involved in this association. Growing evidences point at cerebrovascular alteration, neuroinflammation, and brain insulin resistance as NAFLD/NASH-related CNS manifestations. Since the pharmacological options available for the management of these conditions are still limited, further studies are needed to unravel the mechanism(s) of NAFLD/NASH and their central manifestations and identify effective pharmacological targets.

## 1. Introduction

Non-alcoholic fatty liver disease (NAFLD) is considered the hepatic manifestation of metabolic syndrome and is associated with progressive hepatocellular lipid accumulation, mostly of triglycerides, up to more than 10% of liver weight [1]. This disease comprises a wide range of liver disorders, from simple non-alcoholic fatty liver (NAFL) to non-alcoholic steatohepatitis (NASH) and, if not treated, can lead to threatening complications such as cirrhosis and hepatocellular carcinoma [2]. Generally, NAFLD is considered a benign and reversible condition, although one-third of NAFLD patients eventually progress to NASH, which is characterized by inflammation and hepatocellular injury [3]. While the causes involved in the establishment of NAFLD have been largely investigated, the main factors controlling the progression of NAFLD toward NASH remain pretty much unknown and are currently intensively studied. Many experimental evidences suggested that lipotoxicity, proinflammatory mediators, and oxidative stress may have a central role in this process [3], which can occur in the presence or absence of a high amount of dietary fat ingestion [1]. Moreover, the consumption of imbalanced diets (e.g., excessive fat and sugar intake), as well as the alteration of gut microbiome are involved in NAFLD development and progression [4,5,6]. In particular, high fat and high sugar diets, besides promoting the deposition of fat in the liver, could modify microbiome composition and affect gut barrier integrity, facilitating bacterial translocation and inflammation. Inflammation and oxidative stress have also shown to play a pivotal role in extrahepatic diseases, including many different central nervous system (CNS) diseases such as, for instance, Alzheimer’s disease (AD). Accordingly, extensive evidences obtained in the last years have revealed that NAFLD may represent a risk factor for CNS impairment [7].

Several observations suggest that a correlation may exist between metabolic liver diseases, such as NAFLD and NASH, and CNS disorders, starting from the increased risk of developing AD, mild cognitive impairment (MCI), and dementia in patients with dyslipidemic disorders, and an association with neurodegeneration and cognitive deficits has been observed in patients with metabolic syndrome-related diseases such as type 2 diabetes mellitus (T2DM) and obesity [3].

In this review, we want to point the attention to depression and mild and severe cognitive impairment, which are becoming a serious health threat, and in recent years have been associated to NAFLD and NASH.

## 2. Cognitive Dysfunction and Brain Abnormalities

### 2.1. Depression

Depression is one of the most common CNS diseases involved in adult premature death and it is characterized by specific cognitive and somatic abnormalities over time. The common symptoms of patients with depressive disorders are mood imbalances or anhedonia [8], which are also associated with social dysfunction and cognitive and functional impairment [9].

Since depression is known to be associated with chronic liver disease (CLD) [10,11], and considering that nearly 30% of patients with NAFLD show major depressive disorder (MDD) with a prevalence higher than the general population [12], it is plausible to hypothesize that a correlation exists between NAFLD and depression. However, controversial results have been obtained by the different studies that tried to validate this hypothesis (Table 1). While dated population-based studies reported that NAFLD is absolutely not correlated to depressive disorders [10], other studies conducted in the same period or more recently reported the existence of a possible association between depression and NAFLD [12,13]. In particular, the study of Youssef and collaborators provided robust evidence of the correlation between depressive symptoms and hepatocyte ballooning, one of the main hallmarks of NAFLD progression, even after the adjustment for potential confounding factors such as age, sex, ethnicity, body mass index (BMI), diabetes mellitus, systemic hypertension, smoking, alcohol consumption, and anxiety symptoms. Indeed, a multivariate data analysis showed more severe hepatocyte ballooning in patients with mood disorders, validating the hypothesis of a NAFLD role in depressive symptoms [13]. Accordingly, Tomeno and collaborators suggested a correlation between the severity of steatosis and MDD comorbidity in NAFLD patients. These findings were confirmed by higher levels of serum markers of liver dysfunction such as aspartate aminotransferase (ALT), alanine aminotransferase (AST), γ-glutamyl transpeptidase (GGT), and high-sensitivity C-reactive protein (hs-CRP) in NAFLD patients affected by MDD [12]. Although the results of these pioneering studies need to be confirmed by other investigations, they pointed the attention on the relationship between NAFLD severity and MDD.

For a better understanding of the liver disease influence on depressive disorders, preclinical evaluations were performed in animal models of NASH to verify the presence of behavioral mood changes in these models. The forced swimming test was used in one of these studies to assess depressive-like behavior, like despair and anhedonia, in rats. Rats with NASH showed a lack of struggle to escape, in contrast with their normal attitude. This result demonstrated that NASH rats were affected by a sense of hopelessness, which is normally associated with depression [14]. 

Depression and anxiety have been associated not only with NAFLD, but they have also shown to be strictly involved in pathological features typical of NAFLD progression to NASH, such as insulin resistance and inflammation. The study of Elwing and collaborators tried to evaluate the correlation between these mood disorders and liver histological features. Their findings provide evidence that depression is directly associated with hepatic inflammatory markers, suggesting its active role in NASH progression [11]. One recent study investigated the possible changes of brain tissue volumes in NAFLD patients. They found a significant reduction of white and gray brain volumes and an increased volume of lateral ventricles, with respect to healthy patients. This ulterior remark suggests a higher risk of depression in NAFLD-diagnosed patients [15].

### 2.2. Cognitive Impairment

Mild cognitive impairment (MCI) is defined as an impairment in cognition more severe than that generally associated with normal memory and cognitive changes merely due to aging (clinically considered as “age-related cognitive decline”). However, this condition is less problematic than dementia or other cognitive deficits which significantly impair daily functions [16]. Among the different cognitive features which could be impaired, we will focus on memory, social functioning, visuospatial function, and executive functioning. 

As far as metabolic syndrome and its components are concerned, including liver manifestations as NAFLD/NASH, many recent population-based studies have suggested their involvement in cognitive impairment, from mild ones up to dementia [17]. Although there are studies providing evidences that metabolic syndrome [18,19] and NASH [3,14] are associated with cognitive deficits, whether NAFLD may lead to cognitive impairment remains controversial (Table 2). 

One of the first studies suggesting the presence of functional and cognitive impairment in NAFLD patients was a study by Elliott and collaborators [20]. The main conclusion they achieved was that NAFLD patients had a significantly worse cognitive function with respect to controls, supporting the idea that NAFLD may influence cognitive features.

A lead study in this field has been performed by Seo and collaborators and confirmed the hypothesis of the independent association between NAFLD and cognitive impairment after the analysis of the data obtained from the Third National Health and Nutrition Examination Survey (NHANES III). The authors considered patients with cognitive impairment, excluding possible confounding factors (e.g., BMI, waist circumference, diabetes mellitus, hypertension, hypercholesterolemia, history of acute myocardial infarction, heart failure, and stroke) and considering factors known to affect cognition, such as cardiovascular disease. They observed an increase of liver enzymes, surrogate marker of NAFLD presence and progression to NASH, even without histological signs of advanced liver dysfunction [21].

It is well known that screening tools like the Montreal Cognitive Assessment (MoCA) are quite useful and more sensitive than others for diagnosing forms of cognitive decline in old adults and patients with MCI [22]. Celikbilek and collaborators utilized for the first time the Turkish version of the MoCA test to investigate whether patients with NAFLD show more probability to manifest cognitive impairment than healthy subjects. The results showed a correlation between liver dysfunction and cognitive impairment, in particular, in the visuospatial and executive function domains, both associated with the prefrontal cortex (PFC) [23].

Recent studies tried to assess which brain region(s) may be affected by NAFLD and reported that these patients were characterized by lower levels of metabolic activity in some brain areas, e.g., PFC, hippocampus, and amygdala, due to low levels of dopamine in the PFC and cerebellum and of noradrenaline in the striatum [14]. Taken together, these observations validate the hypothesis of NAFLD implication in cognitive impairment.

Another study investigated the possible correlation between cognitive status and NAFLD using the MoCA test, finding a lower MoCA score and a reduction in white and gray brain volumes in NAFLD patients [15]. In combination with the reduced gray and white volumes, these authors found an increase of lateral ventricles volumes, justifying the constant total brain volume in presence of different cognitive situations between the tested group. The main conclusion achieved by this study is that patients with NAFLD have a risk four times higher of manifesting lower cognitive abilities and depleted cognitive performance and deficit, and also confirmed the higher concentration of AST in NAFLD patients with cognitive deficit [15]. The correlation between higher levels of AST and ALT and poorer cognitive function, especially in visuospatial memory, was also supported by a recent population-based study conducted by An and collaborators [24]. 

Besides these results, there are also studies that found no correlation between NAFLD per se and cognitive impairment, as found in a cross-sectional study by Weinstein et al. [25], who associated poorer cognitive function (mainly in the executive areas) with an increased risk of advanced liver fibrosis but not NAFLD. These results suggest that the association between NAFLD and cognition may be influenced by the specific cognitive brain domains studied and also by the type of liver dysfunction.

Finally, to which extent sleep apnea and chronic intermittent hypoxia, which are known to result in cognitive impairment [26] and also to be associated with the metabolic syndrome and NAFLD/NASH [27], contributes to the cognitive impairment found in NAFLD still remains to be elucidated. 

### 2.3. Neurodegenerative Diseases: Alzheimer’s Disease

Alzheimer’s disease is a neurodegenerative disorder which may derive from MCI progression [22] and it is characterized by the progressive atrophy of cortical and medial temporal structures, CNS areas involved in memory and learning deficits [28]. It belongs to a series of neurodegenerative disease provoking pathophysiological brain changes via accumulation of misfolded proteins, in particular, peptide variants of amyloid-β (Aβ). Progressive protein deposition causes amyloid and senile plaques formation with synaptic dysfunction, dendritic spines loss, and neuronal death [7].

The AD etiology remains unclear, but there are many possible mechanisms, other than aging, proposed to explain its development. Recently, a number of studies provided evidence of a strict correlation between metabolic syndrome-associated diseases, such as diabetes mellitus and NAFLD, and neurodegenerative disorders, like AD [29]. Indeed, de la Monte and collaborators introduced the concept that AD could be considered a neurodegenerative disorder mediated by insulin resistance, since similar abnormalities were found in both pathologies [28]. NAFLD is known to be associated with a dysregulated lipid metabolism and increased cellular oxidative stress, and these same characteristics are present in AD, underlining their possible interconnection. Furthermore, epidemiological data suggested that dyslipidemic and insulin-dependent diseases play a key role as cofactors of AD pathogenesis [3]. 

The hypothesis of a correlation between AD and NAFLD is very recent, and most studies are performed in animal models and not in human patients. One aspect implicated in the development of metabolic syndrome, NAFLD, and potentially AD is the consumption of a high fat diet (HFD). A number of experimental studies used this type of diet in animal models to verify whether an association exists between AD and metabolic syndrome related diseases [30]. One of the first studies conducted in mice chronically fed with HFD showed a time-dependent decline in brain weight with respect to controls. Subtle histopathological abnormalities like neuronal loss foci and cellular apoptosis were also found in brain tissue, pointing to a correlation between HFD-induced NAFLD and mild neuropathological brain lesions [31]. Kim and collaborators evaluated the possible impact of NAFLD in AD pathogenesis, using an AD transgenic mouse model. Their findings suggested an acceleration in neurodegeneration and in Aβ plaque formation after HFD-induced acute inflammation and NAFLD development [30]. The fact that HFD and fructose-rich diets may quicken AD cognitive decline has also been confirmed in recent experimental studies [32,33,34].

A population-based study was carried out to assess whether different biological markers, together with neuropsychological evaluations, could provide a robust method to measure MCI and early AD progression (Table 3). The authors of this study considered different covariates in their evaluations, such as age, sex, BMI, years of education, and APOE ε4 status, and the results they obtained suggested that ALT and AST to ALT ratio, whose levels increased in NAFLD patients, were directly associated with poor cognition and greater Aβ deposition in brain areas [35]. 

Currently, there is a great interest in confirming the existence of a correlation between NAFLD and AD, probably because these diseases are widespread worldwide, and an effective pharmacological treatment is still missing for both of them. New approaches are in the phase of optimization, as suggested by Karbalaei and collaborators, who used a systems biology method to investigate the genes involved in both NAFLD and AD pathophysiological pathways, providing another evidence of their reciprocal interconnection [36].

## 3. Molecular and Pathophysiological Pathways Connecting NAFLD/NASH to Cognitive Impairment

The pathogenesis of NAFLD and NASH is a quite complex process involving multiple pathways and risk factors, first of all being diet imbalances. The progressive lipid deposition in the liver leads to the alteration of lipid metabolism/lipid peroxidation, insulin resistance, oxidative stress, and inflammatory damage. This promotes a state of peripheral insulin resistance and low-grade systemic inflammation. For this reason, an increasing amount of evidence suggests that NAFLD and NASH not only affect liver function, but also induce multiple extrahepatic manifestations that also involve the central nervous system, e.g., depression, cognitive impairment, AD, and dementia. Moreover, emerging evidence has demonstrated the link between microbiome composition and gut impairment, and the development of both liver diseases and cognitive dysfunctions [37,38]. It could be hypothesized that the same detrimental stimuli that lead to NAFLD and NASH in the liver could induce cognitive impairment or AD-type neurodegeneration in the brain. 

Many signaling pathways are demonstrated to be altered in both NAFLD/NASH and CNS dysfunction (depression, cognitive impairment, dementia, and AD), suggesting that these diseases share, at least in part, the same pathogenetic mechanisms. Some studies reported conflicting results and whether there is a causal relation between liver damage and the development of cognitive dysfunction or if steatosis triggers for other subsequent deleterious pathogenetic mechanisms remains to be fully understood [23,39].

**Figure 1:**
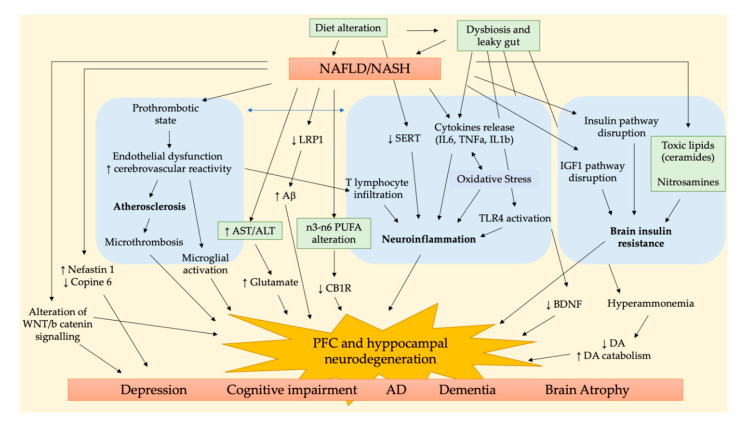
Main pathophysiological pathways involved in NAFLD/NASH and cognitive impairment.

The main pathways involved in the NALFD/NASH-related brain dysfunction are summarized in Figure 1. Three are the main pathological aspects accounted to link NAFLD/NASH to cognitive impairment, i.e., cerebrovascular alteration, neuroinflammation, and brain insulin resistance. Accordingly, the study of Karbalaei et al. suggested three putative groups of genes involved in both AD and NAFLD related to carbohydrate metabolism, long fatty acid metabolism, and interleukin signaling pathways [36]. 

One of the first brain area affected by early stage chronic liver diseases is the cerebellum; then, brain injury could progress to the hippocampus or prefrontal cortex (PFC), brain areas crucial for cognition, memory, learning, and mood regulation [40,41,42,43]. 

The histological analysis of NASH patients’ cerebella revealed the presence of parenchymal microthrombi, neurodegeneration in the Purkinje layer, and glial alteration in the molecular layer, in addition to the activation of microglia and astrocytes of white matter. Some of these features are also observed in some neurodegenerative and vascular diseases, e.g., AD, vascular dementia, and atherosclerosis [44]. 

Petta and collaborators observed in NAFLD patients the presence of cerebral lesions in PFC white matter, whose prevalence increases as liver function worsens, e.g., in NASH and advanced fibrosis, probably due to the proinflammatory and proatherogenic state typical of these advanced stages of liver diseases [45]. This is coherent with the fact that liver steatosis is characterized by a proinflammatory state that promotes atherosclerosis, endothelial dysfunction, platelet, and microglia activation in the brain. These alterations induce micro- and macrovascular damage, which is responsible for clinical and subclinical cerebrovascular dysfunctions [39]. The activation of inflammatory pathways characteristic of NAFLD and NASH induces the production and release of some inflammatory, prothrombotic, and oxidative stress mediators, e.g., the cytokines IL6, TNFα, and IL1β. Moreover, the increased production of reactive oxygen species could sustain the inflammatory cascade, further increasing IL6 release, neuroinflammation, and neurodegeneration [30]. It has been demonstrated that the peripheral inflammation observed in NASH patients may lead to endothelial damage and formation of microthrombi in the brain parenchyma, leading to neuroinflammation. It has been hypothesized that this phenomenon can be due to infiltrating CD4+ T lymphocytes that have been observed in the cerebellar meninges of these patients [44]. In addition, Ghareeb and collaborators observed an alteration in neurotransmitter activities induced by NAFLD, in addition to oxidative stress and metabolic dysfunction, and suggested that this may represent a risk factor for cognitive dysfunction and neurodegeneration [46]. In detail, they observed an increased activity of acetylcholine esterase (AChE) and monoamine oxidase (MAO) in NAFLD liver and brain tissues with respect to controls, accompanied to an increase of ATPase activity, and the inflammatory markers IL6 and TNF-α in the brain.

The Western diet (WD), a diet rich in fat and sugar, is considered one of the main risk factors for NAFLD/NASH, and its implication in the context of neuropsychiatric disorders and cognitive decline is a raising evidence. In the brain, it has been observed that WD is able to increase the activity of the endocannabinoid (eCB) system in the limbic areas controlling emotions, due to the increase of phospholipidic n-6/n-3 PUFA ratio in neuronal membranes and of n-6 PUFAs in the cytoplasm of hippocampal neurons. Cytoplasmatic n-6 PUFAs could be transformed into eCBs, lipid mediators binding to CB1 receptors (CB1R), reducing GABA release from interneurons located in CA1 area and altering theta oscillations. As a consequence of these alterations, WD could lead to an increase vulnerability to neuropsychiatric disorders and cognitive decline [47]. Moreover, an excessive consumption of fructose, a monosaccharide mainly used as sweetening agent in soft drinks, in addition to systemic metabolic alterations, was able to induce oxidative stress, thereby causing lipid peroxidation and protein nitrosylation in the hippocampus and reducing the expression of synaptic proteins, leading to impaired synaptic function, thus affecting learning and memory in a long-lived animal model [48]. Accordingly, many reports investigating the association between obesity and cognitive impairment have reported that the consumption of high-fat and high-sugar diet is able to increase oxidative stress, inflammation, and AChE activity, leading to cerebrovascular changes and neuronal loss in the hippocampus, disrupt myelination and axonal transmission, and decrease of dopamine (DA) and serotonin (5-HT) in the hippocampus, two of the key neurotransmitters involved in learning and memory processes [33,34,49,50]. 

Several pieces of evidence have demonstrated that unhealthy diets, e.g., the Western diet or high fructose intake, could greatly influence gut microbiome composition by decreasing anti-inflammatory autochthonous bacteria and increasing proinflammatory pathological ones. This affects intestinal permeability (“leaky gut”) and leads to an increase of LPS/endotoxin entry into portal and systemic circulation [38]. This process could worsen liver steatosis and induce NASH progression by causing a widespread pro-inflammatory status. CNS function can also be impaired by these events through the gut–brain axis [51,52,53]. These studies further suggest the key role of the consumption of unbalanced diets and gut dysbiosis in the development of NAFLD/NASH, cognitive impairment, and AD.

An interesting review on the influence of liver dysfunction on AD progression suggested that chronic liver diseases may worsen amyloid burden due to an imbalance in peripheral amyloid-β (Aβ) clearance, leading to higher Aβ circulating levels. This can be due to a low hepatic expression of low-density lipoprotein receptor-related protein 1 (LRP-1), necessary for Aβ clearance, consequent to liver dysfunction and chronic inflammation. Furthermore, these features were proposed to negatively affect blood–brain barrier (BBB) integrity, thus contributing to a vicious cycle in Aβ clearance [7]. 

An impaired Wnt/β-catenin signaling pathway was reported to be involved in the pathogenesis of depression and AD [54,55]; thus, the dysregulated expression of proteins of this pathway observed in NAFLD rats could also be responsible for related cognitive impairment [56].

Another study demonstrated that the behavioral and cognitive impairments observed in rats with NAFLD could be linked to an imbalance of nesfatin-1 and copine 6 in the hippocampus and PFC [56]. Nesfatin-1 is able to regulate appetite, glucose, and energy metabolism, but also plays a role in mood and cognitive function [57,58]; thus, its increased plasma levels in NAFLD could be responsible for the observed cognitive dysfunction. Copine 6, a calcium sensor, plays an important role in brain-derived neurotrophic factor (BDNF)-dependent changes in dendritic spines, regulating neurotransmission, and promoting synaptic plasticity, learning and memory [59,60]. A decrease of copine 6 expression was related to depression-like behavior and immune activation and was also observed in NAFLD rats [56,61]. Another hypothesized mechanism leading to an increased risk of dementia and cognitive dysfunction in NAFLD/NASH subjects is related to chronic hyperglycemia and brain insulin resistance. It has been proposed that peripheral insulin resistance could trigger cognitive function through a liver–brain axis of neurodegeneration due to an excessive peripheral production of neurotoxic lipids, e.g., ceramides and nitrosamines, that pass across the blood–brain barrier (BBB) and affect neuronal activity, particularly in the hippocampus and PFC [3]. In the CNS, insulin orchestrates a network of pro-growth and pro-survival signals by activating intracellular pathways, e.g., insulin and insulin-like growth factor type 1 (IGF-1) signaling, thus promoting mitogenesis, cell survival, energy metabolism, and motility. Studies using different experimental models have demonstrated that NASH could increase the hepatic production of ceramides, nitrosamines, and related molecules, causing insulin resistance, oxidative stress, and brain injury. Liver-derived cytotoxic lipids enter the circulation, activate proinflammatory cytokine-mediated injury, and disrupt endothelial cell-to-cell junctions, thus increasing BBB permeability and penetrating in the CNS. Moreover, myelin degradation further increases endogenous ceramide production, exacerbating brain insulin resistance, neuroinflammation, oxidative stress, and neurotransmitter paucity, thus leading to neurocognitive deficits [3,31,62].

Recent evidences have demonstrated a close correlation between the alteration of gut microbiota (dysbiosis) and NASH development due to the increased intestinal permeability to lipopolysaccharide (LPS) that activates Toll-like receptor 4 (TLR4) of hepatic Kupffer cells (KCs) and hepatic stellate cells (HSCs), triggering the pro-inflammatory cytokine cascade that induces and maintains NASH. In the brain, the LPS-activated inflammatory cascade induces a decrease in BDNF expression, reduces the number of viable cells in the pyramidal layer, and promotes neurodegeneration and atrophy of hippocampal neurons, thus affecting CNS function and causing degenerative dementia and cognitive impairment [63]. 

A recent study of Higarza and collaborators demonstrated that a high-fat, high-cholesterol diet induces not only NASH but also dysbiosis, decreasing microbial short chain fatty acid (SCFA) production and increasing ammonia. Since hyperammonemia has been correlated to neuroinflammation and cognitive impairment, these authors suggested that diet-induced dysbiosis disrupts brain metabolism and function, factors contributing to the behavioral deficits observed in NASH. Additionally, they observed an increased DA catabolism in this NASH model, causing a drop of DA levels in the PFC and an increase of DOPA/DA ratio in the cerebellum. This suggests that insulin resistance could lead to dopaminergic dysfunction and a reduction of mitochondrial oxidative activity [14].

## 4. Pharmacological Strategies to Improve NAFLD/NASH-Related Cognitive Impairment

Although the increasing prevalence of NAFLD/NASH has made the need of effective treating options a priority, no therapy for NAFLD patients has been yet approved, and weight loss and increased physical activity remain the two gold standard interventions [64]. Several pharmacological agents have been studied and/or are in the pipeline for their effect on metabolic targets, the anti-inflammatory pathway, or fibrogenesis. Four agents (a PPARα/δ agonist, a FXR agonist, a CCR2/CCR5 antagonist, and an ASK1 inhibitor) are undergoing phase III clinical trials to be evaluated for their ability to reduce insulin resistance and the proinflammatory cascades responsible for NASH progression [65]. In this context, the development of a pharmacological option active also on the brain manifestations of NAFLD/NASH is a great challenge for scientists. Some interventions have been proposed in the last few years; nevertheless, their real usefulness as clinical pharmacological treatments needs to be further demonstrated.

Since insulin resistance is a distinctive feature of both steatohepatitis and AD, some studies have proposed the use of insulin-sensitizing agents, such as PPAR agonists, that are able to activate insulin-responsive genes and their signaling pathways to treat liver and brain insulin resistance-mediated diseases [66]. Early treatment with PPAR agonists has shown to effectively prevent brain atrophy, neurodegeneration, and its associated learning and memory impairment, preserving neurons expressing the insulin receptor and IGF receptor and maintaining cholinergic homeostasis and myelin expression [67,68]. Their antioxidant activity in the CNS was also reported to further sustain their therapeutic use in the content of oxidative stress-related diseases such as NAFLD, NASH, and AD. 

Other studies demonstrated that in early stage AD patients, an improvement or stabilization of their cognitive impairment was obtained with intranasal insulin administration, leading to increased brain insulin levels [69,70,71,72]. 

Some studies have reported that chromium picolinate is able to improve insulin sensitivity by reducing glucose and insulin levels in overweight or obese subjects, and to increase HDL cholesterol and decrease LDL levels, thus controlling metabolic syndrome risk factors [73,74]. Moreover, this agent could improve cognitive function in elderly people, reducing semantic interference on learning, recall, and memory [75,76]. These positive effects on the insulin signaling pathway, both at the systemic and central level, suggest the use of chromium picolinate as a dietary supplement in NAFLD-related cognitive impairment and AD, even though clinical studies need to be performed to support this hypothesis.

The use of dietary supplements is widely exploited to support the dietary interventions in patients with metabolic syndrome, obesity, and NAFLD. A recent study on resveratrol, a polyphenol naturally present in grapes, blueberries, raspberries, and mulberries, has reported the improvement of both liver metabolic dysfunction and behavioral and cognitive impairments in a rat model of NAFLD [77]. Resveratrol administration to NAFLD rats is able to ameliorate the imbalanced expression of copine 6, p-catenin, and p-GSK3β in the hippocampus and PFC, restoring normal protein levels and improving the altered Wnt/β-catenin signaling pathway. This study further supports previous evidences on the effect of resveratrol in reducing Aβ plaque formation associated with AD [78].

Another study investigating the effect of *Curcuma* derivatives demonstrated their ability in improving high-fat and high-sugar diet-induced obesity, oxidative stress, memory impairment, and neurodegeneration. These effects have been attributed to the antioxidant properties and anticholinesterase activity of curcuminoids and terpenoids present in the tested extract. Furthermore, they increased serotonin and dopamine levels, exerting neuroprotection of hippocampal neurons [79].

Since several pieces of evidence suggested that liver steatosis may negatively affect cognitive performance in AD subjects, n3-PUFA supplementation can be considered as an option to improve NAFLD-related brain dysfunction, since these fatty acids are able to improve liver n3/n6 PUFA imbalance and modulate many neuronal functions, protecting them from oxidative stress and inhibiting signaling pathways responsible for tau phosphorylation in AD and dementia patients [80].

As stated before, the gut–brain axis and dysbiosis could play a role in the cognitive impairment of NAFLD and NASH patients. Starting from the observation that probiotics have demonstrated to improve NASH by decreasing LPS-induced pro-inflammatory cytokines, such as IL6 and TNF-α [81], a study of Mohammed et al. investigated the effect of *Lactobacillus plantarum* (*LP EMCC-1039*) administration on cognitive performance and liver function in dysbiosis-induced NASH in rats. *LP EMCC-1039* supplementation was correlated to an improvement of cognitive function in these animals due to the modulation of TLR4/BDNF signaling pathway, and an increase of viable cells and of the thickness of the pyramidal layer was observed [63]. 

## 5. Conclusions

In conclusion, increasing evidences suggest that a correlation exists between NAFLD/NASH and CNS diseases or dysfunctions such as depression, MCI, AD, and dementia. Growing evidences point at cerebrovascular alteration, neuroinflammation, and brain insulin resistance as NAFLD/NASH-related CNS manifestations. Unfortunately, the pharmacological options available for the management of these conditions are still limited, both in number and in efficacy. Further experimental and clinical studies are needed to gain new insights about the mechanism(s) of NAFLD/NASH and their central manifestations and identify effective pharmacological targets, after the comprehension of the complex mechanisms involved in the NAFLD/NASH, including the regulation of the gut–brain axis by diet and microbiome composition.

## Figures and Tables

**Table 1:** Principal clinical studies reporting an association between depressive disorders and NAFLD.

Study	Settings and Study Design	Subjects	Methods	Results and Conclusions
Lee et al. (2013) [10]	Cross-sectional national survey, population-based	10231 NHANES participants in the 18th year or older	PHQ-9 survey to screen depression associated with hematologic and biochemical tests and viral hepatitis	Depression and chronic hepatitis C are independently associated, but not metabolic syndrome
				
Tomeno et al. (2015) [12]	Population-based	258 participants	Blood test monitoring and lifestyle counseling for 48 weeks, with assessment of insulin resistance through HOMA-IR	32 NAFLD patients were comorbid with MDD and showed higher biochemical parameters (ALT, AST, GGT, ferritin, hs-CRP, and cholinesterase) than NAFLD patients without MDD. Only NAFLD patients without MDD improved their conditions with treatment.
				
Youssef et al. (2013) [13]	Cross-sectional analyses, population-based	567 participants aged 20 and older	HADS questionnaire to assess severity of depression and anxiety	Severe depressive symptoms were associated with increased hepatocyte ballooning
				
Elwing et al. (2006) [11]	Case-control comparison	36 patients undergoing cholecystectomy and 36 matched control subjects	Structured interview to assess psychiatric illnesses	Lifetime MDD has significantly increased rates in NASH subjects, in accordance with PHQ-9.
				
Filipović et al. (2018) [15]	Population-based	40 NAFLD positive participants aged from 34 to 57, and 36 controls aged from 39 to 53	3D T1-weighted MR images to measure gray and white matter volume and brain lateral ventricles, Serbian version of the MoCA test to assess cognitive functioning and Hamilton’s depression rating scale to evaluate depression level	Cognitive status declined in NAFLD patients, according to the MoCA index. These patients had reduced gray and white matter volumes and higher risk of depression.

NHANES: National Health and Nutrition Examination Survey; PHQ-9: Patient Health Questionnaire; HOMA-IR: Homeostasis Model for the Assessment of Insulin Resistance; HADS: Hospital Anxiety & Depression Scale; MoCA: Montreal Cognitive Assessment.

**Table 2:** Principal clinical studies reported on cognitive impairment, MCI, and NAFLD patients.

Study	Settings and Study Design	Subjects	Methods	Results and Conclusions
Elliott et al. (2013) [20]	Cohort study	224 NAFLD participants and 100 controls	PHAQ and CFQ were used to evaluate functional and physical ability and cognitive abilities.	NAFLD patients showed significantly worse functional abilities, and they had more difficulties in specific daily activities than controls.
				
Seo et al. (2016) [21]	Cross-sectional population-based analysis	4472 participants aged from 20 to 59	Assessment of liver enzyme activity and cognitive evaluation using SRTT, SDLT, and SDST	NAFLD patients showed lower performance on the SDLT, and NAFLD resulted independently associated with lower cognitive performance.
				
Celikbilek et al. (2018) [23]	Prospective cross-sectional population-based analysis	70 participants and 73 age- and sex-matched controls aged from 18 to 70	Turkish version of the MoCA test to evaluate cognitive functions	Deficits were observed in each cognitive domain, mainly in the visuospatial and executive functioning. NAFLD patients reported significantly lower MoCA test scores.
				
An et al. (2019) [24]	Cross-sectional population-based analysis	23 NAFLD participants and 21 matched controls	BDI was used to assess depressive symptoms, and RBANS was used to characterize neurocognitive deficits.	BDI mean score indicated a moderate depression in NAFLD patients, and women reported significant association with visuospatial memory deficit.
				
Weinstein et al. (2019) [25]	Cross-sectional population-based analysis	1287 participants	Trail-making test to measure executive functioning. Similarity test was used to assess abstract reasoning skills, and the Hooper visual organization test was used to measure visual perception.	NAFLD and cognitive performances were not associated; however, poorer performances on the trail-making and similarities tests were linked to increased risk of advanced fibrosis in NAFLD participants.

PHAQ: Patient-Reported Outcomes Measurement Information System, Health Assessment Questionnaire; CFQ: Cognitive Failures Questionnaire; SRTT: Simple Reaction Time Test; SDLT: Serial Digit Learning Test; SDST: Symbol-Digit Substitution Test; BDI: Beck Depression Inventory; RBANS: Repeatable Battery for Assessment of Neuropsychological Status.

**Table 3:** Principal clinical study reported on the Alzheimer’s disease and NAFLD connection.

Study	Settings and Study Design	Subjects	Methods	Results and Conclusions
Nho et al. (2019) [35]	Cohort study	1581 participants aged around 70	Evaluation of cerebrospinal fluid biomarkers and brain atrophy (magnetic resonance), and scores for executive functioning and memory	Increased ALT and AST to ALT ratio in AD patients were linked to poor cognition.

## References

[1] Engin A., Engin A.B., Engin A. (2017). Non-Alcoholic Fatty Liver Disease. Obesity and Lipotoxicity.

[2] Ekstedt M., Nasr P., Kechagias S. (2017). Natural History of NAFLD/NASH. Curr. Hepatol. Rep..

[3] De la Monte S.M., Longato L., Tong M., Wands J.R. (2009). Insulin resistance and neurodegeneration: Roles of obesity, type 2 diabetes mellitus and non-alcoholic steatohepatitis. Curr. Opin. Investig. Drugs.

[4] Elshaghabee F.M.F., Rokana N., Panwar H., Heller K.J., Schrezenmeir J. (2019). Probiotics for dietary management of non-alcoholic fatty liver disease. Environ. Chem. Lett..

[5] Gabbia D., Roverso M., Guido M., Sacchi D., Scaffidi M., Carrara M., Orso G., Russo F.P., Floreani A., Bogialli S. (2019). Western Diet-Induced Metabolic Alterations Affect Circulating Markers of Liver Function before the Development of Steatosis. Nutrients.

[6] Gabbia D., Saponaro M., Sarcognato S., Guido M., Ferri N., Carrara M., De Martin S. (2020). Fucus vesiculosus and Ascophyllum nodosum Ameliorate Liver Function by Reducing Diet-Induced Steatosis in Rats. Mar. Drugs.

[7] Estrada L.D., Ahumada P., Cabrera D., Arab J.P. (2019). Liver Dysfunction as a Novel Player in Alzheimer’s Progression: Looking Outside the Brain. Front. Aging Neurosci..

[8] Kaltenboeck A., Harmer C. (2018). The neuroscience of depressive disorders: A brief review of the past and some considerations about the future. Brain Neurosci. Adv..

[9] Gonda X., Pompili M., Serafini G., Carvalho A.F., Rihmer Z., Dome P. (2015). The role of cognitive dysfunction in the symptoms and remission from depression. Ann. Gen. Psychiatry.

[10] Lee K., Otgonsuren M., Younoszai Z., Mir H.M., Younossi Z.M. (2013). Association of Chronic Liver Disease with Depression: A Population-Based Study. Psychosomatics.

[11] Elwing J.E., Lustman P.J., Wang H.L., Clouse R.E. (2006). Depression, Anxiety, and Nonalcoholic Steatohepatitis. Psychosom. Med..

[12] Tomeno W., Kawashima K., Yoneda M., Saito S., Ogawa Y., Honda Y., Kessoku T., Imajo K., Mawatari H., Fujita K. (2015). Non-alcoholic fatty liver disease comorbid with major depressive disorder: The pathological features and poor therapeutic efficacy: Fatty liver comorbid with depression. J. Gastroenterol. Hepatol..

[13] Youssef N.A., Abdelmalek M.F., Binks M., Guy C.D., Omenetti A., Smith A.D., Diehl A.M.E., Suzuki A. (2013). Associations of depression, anxiety and antidepressants with histological severity of nonalcoholic fatty liver disease. Liver Int..

[14] Higarza S.G., Arboleya S., Gueimonde M., Gómez-Lázaro E., Arias J.L., Arias N. (2019). Neurobehavioral dysfunction in non-alcoholic steatohepatitis is associated with hyperammonemia, gut dysbiosis, and metabolic and functional brain regional deficits. PLoS ONE.

[15] Filipović B., Marković O., Đurić V., Filipović B. (2018). Cognitive Changes and Brain Volume Reduction in Patients with Nonalcoholic Fatty Liver Disease. Can. J. Gastroenterol. Hepatol..

[16] Sanford A.M. (2017). Mild Cognitive Impairment. Clin. Geriatr. Med..

[17] Celikbilek A., Celikbilek M. (2020). Cognitive impairment in patients with nonalcoholic fatty liver disease with liver fibrosis. Liver Int..

[18] Panza F., Frisardi V., Seripa D., P Imbimbo B., Sancarlo D., D’Onofrio G., Addante F., Paris F., Pilotto A., Solfrizzi V. (2011). Metabolic Syndrome, Mild Cognitive Impairment and Dementia. CAR.

[19] Levin B.E., Llabre M.M., Dong C., Elkind M.S.V., Stern Y., Rundek T., Sacco R.L., Wright C.B. (2014). Modeling Metabolic Syndrome and Its Association with Cognition: The Northern Manhattan Study. J. Int. Neuropsychol. Soc..

[20] Elliott C., Frith J., Day C.P., Jones D.E.J., Newton J.L. (2013). Functional Impairment in Alcoholic Liver Disease and Non-alcoholic Fatty Liver Disease Is Significant and Persists over 3 Years of Follow-Up. Dig. Dis. Sci..

[21] Seo S.W., Gottesman R.F., Clark J.M., Hernaez R., Chang Y., Kim C., Ha K.H., Guallar E., Lazo M. (2016). Nonalcoholic fatty liver disease is associated with cognitive function in adults. Neurology.

[22] Jongsiriyanyong S., Limpawattana P. (2018). Mild Cognitive Impairment in Clinical Practice: A Review Article. Am. J. Alzheimers Dis. Other Demen..

[23] Celikbilek A., Celikbilek M., Bozkurt G. (2018). Cognitive assessment of patients with nonalcoholic fatty liver disease. Eur. J. Gastroenterol. Hepatol..

[24] An K., Starkweather A., Sturgill J., Salyer J., Sterling R.K. (2019). Association of CTRP13 With Liver Enzymes and Cognitive Symptoms in Nonalcoholic Fatty Liver Disease. Nurs. Res..

[25] Weinstein G., Davis-Plourde K., Himali J.J., Zelber-Sagi S., Beiser A.S., Seshadri S. (2019). Non-alcoholic fatty liver disease, liver fibrosis score and cognitive function in middle-aged adults: The Framingham Study. Liver Int..

[26] Vanek J., Prasko J., Genzor S., Ociskova M., Kantor K., Holubova M., Slepecky M., Nesnidal V., Kolek A., Sova M. (2020). Obstructive sleep apnea, depression and cognitive impairment. Sleep Med..

[27] Parikh M.P., Gupta N.M., McCullough A.J. (2019). Obstructive Sleep Apnea and the Liver. Clin. Liver Dis..

[28] De la Monte S.M. (2017). Insulin Resistance and Neurodegeneration: Progress Towards the Development of New Therapeutics for Alzheimer’s Disease. Drugs.

[29] De la Monte S.M., Tong M. (2014). Brain metabolic dysfunction at the core of Alzheimer’s disease. Biochem. Pharm..

[30] Kim D.-G., Krenz A., Toussaint L.E., Maurer K.J., Robinson S.-A., Yan A., Torres L., Bynoe M.S. (2016). Non-alcoholic fatty liver disease induces signs of Alzheimer’s disease (AD) in wild-type mice and accelerates pathological signs of AD in an AD model. J. Neuroinflamm..

[31] Lyn-Cook L.E., Lawton M., Tong M., Silbermann E., Longato L., Jiao P., Mark P., Wands J.R., Xu H., de la Monte S.M. (2009). Hepatic ceramide may mediate brain insulin resistance and neurodegeneration in type 2 diabetes and non-alcoholic steatohepatitis. J. Alzheimers Dis..

[32] Pinçon A., De Montgolfier O., Akkoyunlu N., Daneault C., Pouliot P., Villeneuve L., Lesage F., Levy B.I., Thorin-Trescases N., Thorin É. (2019). Non-Alcoholic Fatty Liver Disease, and the Underlying Altered Fatty Acid Metabolism, Reveals Brain Hypoperfusion and Contributes to the Cognitive Decline in APP/PS1 Mice. Metabolites.

[33] Beilharz J.E., Maniam J., Morris M.J. (2015). Diet-Induced Cognitive Deficits: The Role of Fat and Sugar, Potential Mechanisms and Nutritional Interventions. Nutrients.

[34] Guimarães C.A., Biella M.S., Lopes A., Deroza P.F., Oliveira M.B., Macan T.P., Streck E.L., Ferreira G.C., Zugno A.I., Schuck P.F. (2014). In vivo and in vitro effects of fructose on rat brain acetylcholinesterase activity: An ontogenetic study. Acad. Bras. Cienc..

[35] Nho K., Kueider-Paisley A., Ahmad S., MahmoudianDehkordi S., Arnold M., Risacher S.L., Louie G., Blach C., Baillie R., Han X. (2019). Association of Altered Liver Enzymes With Alzheimer Disease Diagnosis, Cognition, Neuroimaging Measures, and Cerebrospinal Fluid Biomarkers. JAMA Netw. Open.

[36] Karbalaei R., Allahyari M., Rezaei-Tavirani M., Asadzadeh-Aghdaei H., Zali M.R. (2018). Protein-protein interaction analysis of Alzheimer’s disease and NAFLD based on systems biology methods unhide common ancestor pathways. Gastroenterol. Hepatol. Bed. Bench..

[37] Hu X., Wang T., Jin F. (2016). Alzheimer’s disease and gut microbiota. Sci. China Life Sci..

[38] Fukui H. (2019). Role of Gut Dysbiosis in Liver Diseases: What Have We Learned So Far?. Diseases.

[39] Lombardi R., Fargion S., Fracanzani A.L. (2019). Brain involvement in non-alcoholic fatty liver disease (NAFLD): A systematic review. Dig. Liver Dis..

[40] Felipo V., Ordoño J.F., Urios A., El Mlili N., Giménez-Garzó C., Aguado C., González-Lopez O., Giner-Duran R., Serra M.A., Wassel A. (2012). Patients with minimal hepatic encephalopathy show impaired mismatch negativity correlating with reduced performance in attention tests. Hepatology.

[41] Felipo V., Urios A., Giménez-Garzó C., Cauli O., Andrés-Costa M.-J., González O., Serra M.A., Sánchez-González J., Aliaga R., Giner-Durán R. (2014). Non invasive blood flow measurement in cerebellum detects minimal hepatic encephalopathy earlier than psychometric tests. World J. Gastroenterol..

[42] Butz M., Timmermann L., Braun M., Groiss S.J., Wojtecki L., Ostrowski S., Krause H., Pollok B., Gross J., Südmeyer M. (2010). Motor impairment in liver cirrhosis without and with minimal hepatic encephalopathy. Acta Neurol. Scand..

[43] Giménez-Garzó C., Garcés J.J., Urios A., Mangas-Losada A., García-García R., González-López O., Giner-Durán R., Escudero-García D., Serra M.A., Soria E. (2017). The PHES battery does not detect all cirrhotic patients with early neurological deficits, which are different in different patients. PLoS ONE.

[44] Balzano T., Forteza J., Borreda I., Molina P., Giner J., Leone P., Urios A., Montoliu C., Felipo V. (2018). Histological Features of Cerebellar Neuropathology in Patients With Alcoholic and Nonalcoholic Steatohepatitis. J. Neuropathol. Exp. Neurol..

[45] Petta S., Tuttolomondo A., Gagliardo C., Zafonte R., Brancatelli G., Cabibi D., Cammà C., Di Marco V., Galvano L., La Tona G. (2016). The Presence of White Matter Lesions Is Associated With the Fibrosis Severity of Nonalcoholic Fatty Liver Disease. Medicine (Baltimore).

[46] Ghareeb D.A., Hafez H.S., Hussien H.M., Kabapy N.F. (2011). Non-alcoholic fatty liver induces insulin resistance and metabolic disorders with development of brain damage and dysfunction. Metab. Brain Dis..

[47] Dagnino-Subiabre A. (2019). Stress and Western diets increase vulnerability to neuropsychiatric disorders: A common mechanism. Nutr. Neurosci..

[48] Rivera D.S., Lindsay C.B., Codocedo J.F., Carreño L.E., Cabrera D., Arrese M.A., Vio C.P., Bozinovic F., Inestrosa N.C. (2018). Long-Term, Fructose-Induced Metabolic Syndrome-Like Condition Is Associated with Higher Metabolism, Reduced Synaptic Plasticity and Cognitive Impairment in Octodon degus. Mol. Neurobiol..

[49] Singh D.P., Kondepudi K.K., Bishnoi M., Chopra K. (2014). Altered Monoamine Metabolism in High Fat Diet Induced Neuropsychiatric Changes in Rats. J. Obes. Weight Loss Ther..

[50] Castellani G., Contarini G., Mereu M., Albanesi E., Devroye C., D’Amore C., Ferretti V., De Martin S., Papaleo F. (2019). Dopamine-mediated immunomodulation affects choroid plexus function. Brain Behav. Immun..

[51] Paik Y.-H., Schwabe R.F., Bataller R., Russo M.P., Jobin C., Brenner D.A. (2003). Toll-Like receptor 4 mediates inflammatory signaling by bacterial lipopolysaccharide in human hepatic stellate cells. Hepatology.

[52] Baothman O.A., Zamzami M.A., Taher I., Abubaker J., Abu-Farha M. (2016). The role of Gut Microbiota in the development of obesity and Diabetes. Lipids Health Dis..

[53] Takeda S., Sato N., Morishita R. (2014). Systemic inflammation, blood-brain barrier vulnerability and cognitive/non-cognitive symptoms in Alzheimer disease: Relevance to pathogenesis and therapy. Front. Aging Neurosci..

[54] Folke J., Pakkenberg B., Brudek T. (2019). Impaired Wnt Signaling in the Prefrontal Cortex of Alzheimer’s Disease. Mol. Neurobiol..

[55] Xu L.-Z., Xu D.-F., Han Y., Liu L.-J., Sun C.-Y., Deng J.-H., Zhang R.-X., Yuan M., Zhang S.-Z., Li Z.-M. (2017). BDNF-GSK-3β-β-Catenin Pathway in the mPFC Is Involved in Antidepressant-Like Effects of Morinda officinalis Oligosaccharides in Rats. Int. J. Neuropsychopharmacol..

[56] Chen Z., Xu Y.-Y., Wu R., Han Y.-X., Yu Y., Ge J.-F., Chen F.-H. (2017). Impaired learning and memory in rats induced by a high-fat diet: Involvement with the imbalance of nesfatin-1 abundance and copine 6 expression. J. Neuroendocr..

[57] Ge J.-F., Xu Y.-Y., Qin G., Peng Y.-N., Zhang C.-F., Liu X.-R., Liang L.-C., Wang Z.-Z., Chen F.-H. (2015). Depression-like Behavior Induced by Nesfatin-1 in Rats: Involvement of Increased Immune Activation and Imbalance of Synaptic Vesicle Proteins. Front. Neurosci..

[58] Ge J.-F., Xu Y.-Y., Qin G., Pan X.-Y., Cheng J.-Q., Chen F.-H. (2015). Nesfatin-1, a potent anorexic agent, decreases exploration and induces anxiety-like behavior in rats without altering learning or memory. Brain Res..

[59] Reinhard J.R., Kriz A., Galic M., Angliker N., Rajalu M., Vogt K.E., Ruegg M.A. (2016). The calcium sensor Copine-6 regulates spine structural plasticity and learning and memory. Nat. Commun..

[60] Burk K., Ramachandran B., Ahmed S., Hurtado-Zavala J.I., Awasthi A., Benito E., Faram R., Ahmad H., Swaminathan A., McIlhinney J. (2018). Regulation of Dendritic Spine Morphology in Hippocampal Neurons by Copine-6. Cereb. Cortex..

[61] Han Y.-X., Tao C., Gao X.-R., Wang L., Jiang F.-H., Wang C., Fang K., Chen X.-X., Chen Z., Ge J.-F. (2018). BDNF-Related Imbalance of Copine 6 and Synaptic Plasticity Markers Couples With Depression-Like Behavior and Immune Activation in CUMS Rats. Front. Neurosci..

[62] Tong M., Neusner A., Longato L., Lawton M., Wands J.R. (2010). Nitrosamine Exposure Causes Insulin Resistance Diseases: Relevance to Type 2 Diabetes Mellitus, Non-Alcoholic Steatohepatitis, and Alzheimer’s Disease. J. Alzheimer’s Dis..

[63] Mohammed S.K., Magdy Y.M., El-Waseef D.A., Nabih E.S., Hamouda M.A., El-Kharashi O.A. (2020). Modulation of hippocampal TLR4/BDNF signal pathway using probiotics is a step closer towards treating cognitive impairment in NASH model. Physiol. Behav..

[64] Younossi Z.M. (2019). Non-alcoholic fatty liver disease—A global public health perspective. J. Hepatol..

[65] Alkhouri N., Scott A. (2018). An update on the pharmacological treatment of nonalcoholic fatty liver disease: Beyond lifestyle modifications. Clin. Liver Dis..

[66] De la Monte S.M. (2012). Brain Insulin Resistance and Deficiency as Therapeutic Targets in Alzheimer’s Disease. Curr. Alzheimer Res..

[67] De la Monte S.M., Tong M., Lester-Coll N., Plater J., Wands J.R. (2006). Therapeutic rescue of neurodegeneration in experimental type 3 diabetes: Relevance to Alzheimer’s disease. J. Alzheimer’s Dis..

[68] Landreth G. (2007). Therapeutic use of agonists of the nuclear receptor PPARgamma in Alzheimer’s disease. Curr. Alzheimer Res..

[69] Reger M.A., Watson G.S., Frey W.H., Baker L.D., Cholerton B., Keeling M.L., Belongia D.A., Fishel M.A., Plymate S.R., Schellenberg G.D. (2006). Effects of intranasal insulin on cognition in memory-impaired older adults: Modulation by APOE genotype. Neurobiol. Aging.

[70] Benedict C., Hallschmid M., Hatke A., Schultes B., Fehm H.L., Born J., Kern W. (2004). Intranasal insulin improves memory in humans. Psychoneuroendocrinology.

[71] Benedict C., Hallschmid M., Schmitz K., Schultes B., Ratter F., Fehm H.L., Born J., Kern W. (2007). Intranasal insulin improves memory in humans: Superiority of insulin aspart. Neuropsychopharmacology.

[72] Watson G.S., Cholerton B.A., Reger M.A., Baker L.D., Plymate S.R., Asthana S., Fishel M.A., Kulstad J.J., Green P.S., Cook D.G. (2005). Preserved cognition in patients with early Alzheimer disease and amnestic mild cognitive impairment during treatment with rosiglitazone: A preliminary study. Am. J. Geriatr. Psychiatry.

[73] De Martin S., Gabbia D., Carrara M., Ferri N. (2018). The Brown Algae Fucus vesiculosus and Ascophyllum nodosum Reduce Metabolic Syndrome Risk Factors: A Clinical Study. Nat. Prod. Commun..

[74] Havel P.J. (2004). A scientific review: The role of chromium in insulin resistance. Diabetes Educ..

[75] Krikorian R., Eliassen J.C., Boespflug E.L., Nash T.A., Shidler M.D. (2010). Improved cognitive-cerebral function in older adults with chromium supplementation. Nutr. Neurosci..

[76] Smorgon C., Mari E., Atti A.R., Dalla Nora E., Zamboni P.F., Calzoni F., Passaro A., Fellin R. (2004). Trace elements and cognitive impairment: An elderly cohort study. Arch. Gerontol. Geriatr..

[77] Chen X.-X., Xu Y.-Y., Wu R., Chen Z., Fang K., Han Y.-X., Yu Y., Huang L.-L., Peng L., Ge J.-F. (2019). Resveratrol Reduces Glucolipid Metabolic Dysfunction and Learning and Memory Impairment in a NAFLD Rat Model: Involvement in Regulating the Imbalance of Nesfatin-1 Abundance and Copine 6 Expression. Front. Endocrinol..

[78] Karuppagounder S.S., Pinto J.T., Xu H., Chen H.-L., Beal M.F., Gibson G.E. (2009). Dietary supplementation with resveratrol reduces plaque pathology in a transgenic model of Alzheimer’s disease. Neurochem. Int..

[79] Rao L.S.N., Kilari E.K., Kola P.K. (2019). Protective effect of Curcuma amada acetone extract against high-fat and high-sugar diet-induced obesity and memory impairment. Nutr. Neurosci..

[80] Cole G.M., Ma Q.-L., Frautschy S.A. (2009). Omega-3 fatty acids and dementia. Prostaglandins Leukot. Essent. Fat. Acids.

[81] Medina J., Fernández-Salazar L.I., García-Buey L., Moreno-Otero R. (2004). Approach to the pathogenesis and treatment of nonalcoholic steatohepatitis. Diabetes Care.

